# Testing of Multifractional Brownian Motion

**DOI:** 10.3390/e22121403

**Published:** 2020-12-12

**Authors:** Michał Balcerek, Krzysztof Burnecki

**Affiliations:** Faculty of Pure and Applied Mathematics, Hugo Steinhaus Center, Wroclaw University of Science and Technology, Wyspianskiego 27, 50-370 Wroclaw, Poland; krzysztof.burnecki@pwr.edu.pl

**Keywords:** multifractional Brownian motion, autocovariance function, power of the statistical test, Monte Carlo simulations

## Abstract

Fractional Brownian motion (FBM) is a generalization of the classical Brownian motion. Most of its statistical properties are characterized by the self-similarity (Hurst) index 0<H<1. In nature one often observes changes in the dynamics of a system over time. For example, this is true in single-particle tracking experiments where a transient behavior is revealed. The stationarity of increments of FBM restricts substantially its applicability to model such phenomena. Several generalizations of FBM have been proposed in the literature. One of these is called multifractional Brownian motion (MFBM) where the Hurst index becomes a function of time. In this paper, we introduce a rigorous statistical test on MFBM based on its covariance function. We consider three examples of the functions of the Hurst parameter: linear, logistic, and periodic. We study the power of the test for alternatives being MFBMs with different linear, logistic, and periodic Hurst exponent functions by utilizing Monte Carlo simulations. We also analyze mean-squared displacement (MSD) for the three cases of MFBM by comparing the ensemble average MSD and ensemble average time average MSD, which is related to the notion of ergodicity breaking. We believe that the presented results will be helpful in the analysis of various anomalous diffusion phenomena.

## 1. Introduction

Over the last decades, massive advances in single-particle tracking (SPT), partially based on superresolution microscopy of fluorescently tagged tracers, or fluorescence correlation spectroscopy allow experimentalists to obtain insight into the motion of submicron tracer particles or even single molecules in complex environments, such as living biological cells, down to nanometer precision and at submillisecond time resolution [1,2].

The observed data obtained by SPT experiments often show pronounced deviations from Brownian motion, namely, anomalous diffusion of the power-law form

(1) $$EX{\left(t\right)}^{2}≃K_{\alpha }t^{\alpha }$$

of the mean-squared displacement (MSD) is observed [3,4,5,6,7,8]. $K_{\alpha }$ is the anomalous diffusion coefficient. Depending on the magnitude of the anomalous diffusion exponent α we distinguish subdiffusion for 0<α<1 from superdiffusion with α>1 [5,8]. Subdiffusion is typically observed for submicron particles in both bacterial and eukaryotic cells [6,9,10,11,12,13], in artificially crowded [14,15] and structured [16,17,18,19] liquids, in pure and protein-crowded lipid bilayer systems [12,20,21,22,23,24,25], as well as in groundwater systems [26]. Superdiffusion occurs in the presence of active motion, for instance, in living biological cells [27,28,29] or due to bulk-surface exchange [30,31]. While typically anomalous diffusion refers to the power-law behavior (1) with a fixed α, an increasing number of systems are reported in which the local scaling exponent of the MSD (1) is an explicit function of time, α(t). Such transient behavior has, for instance, been observed for green fluorescent proteins in cells or for the motion of lipid molecules in protein-crowded bilayer membranes [25,32].

Fractional Brownian motion (FBM) introduced by Kolmogorov in 1940 and rediscovered by Mandelbrot and van Ness in 1968 [33,34,35] is a generalization of the classical Brownian motion (BM). Most of its statistical properties are characterized by the self-similarity (Hurst) index 0<H<1. FBM is *H*-self-similar, namely for every c>0 we have $B_{H}\left(ct\right)\overset{D}{=}c^{H}B_{H}\left(t\right)$ in the sense of all finite dimensional distributions, and has stationary increments. It is the only Gaussian process satisfying these properties. FBM is the overdamped description for viscoelastic motion and thus intimately connected to the fractional Langevin processes, an attractive framework for many physical systems [36], for instance, of lipid molecules in bilayer membranes [22,25,37]. The second moment of FBM reads $EB_{H}^{2}\left(t\right)=\sigma^{2}t^{2H}$, where $EB_{H}^{2}\left(1\right)=\sigma^{2}>0$. As a consequence, for H<1/2 we obtain subdiffusive dynamics with persistent motion, whereas for H>1/2, the process is superdiffusive and antipersistent. Since FBM is the classical model for power-law dependence a number of statistical tests have been already introduced for this process in the literature. Let us mention here the tests based on the autocovariance function (ACVF), MSD, and detrending moving average statistics [38,39,40].

FBM has stationary increments that do not allow us to model processes whose regularity of paths and “memory depth” change in time [41]. Several generalizations of FBM have been proposed recently. One of these, called multifractional Brownian motion (MFBM), was proposed by Peltier and Véhel [42] with time-varying Hurst exponent H(t) which is a Hölder function. The variance at time *t* of the MFBM $B_{H(t)}\left(t\right)$ is given by $\mathrm{Var}\left(B_{H(t)}\left(t\right)\right)=\sigma^{2}t^{2H(t)}$ [43]. The time-varying Hurst exponent H(t) characterizes the path regularity of the process at time *t*: sample paths near *t* with small $H_{t}$, close to 0, are space-filling and highly irregular, while paths with large $H_{t}$, close to 1, are very smooth. The variance constant $\sigma^{2}$ determines the “energy level” of the process. This natural extension of FBM results in some loss of some of FBMs basic properties, in particular, the increments of MFBM are non-stationary and the process is no longer self-similar.

Other, similar generalizations are limited to a piecewise constant *H* [44] but, what is important from a data analysis point of view, is that they lead to continuous Gaussian processes with stationary increments. Let us also mention an idea involving an appropriate class of covariance functions. Ryvkina [45] uses such covariance functions to define Gaussian processes to extend FBM and MFBM to a class of fractional Brownian motions with a variable Hurst parameter parameterized by a set of all measurable functions with values in (1/2,1), and different from MFBMs. However, from a biological data point of view, such a range for *H* values is not practical since it only corresponds to a superdiffusive (long-range dependent) case.

MFBMs have become popular as flexible models in describing real-life signals of high-frequency features in geoscience, microeconomics, and turbulence, to name a few [43]. They are closely related to the notion of transient diffusion dynamics observed in biological experiments. The article is structured as follows. In Section 2, the MFBM is defined and its basic properties are presented. We also recall formulas for the ensemble average MSD, time average MSD, and present three Hurst exponent functions that will be analyzed in the sequel. In Section 3, the main results are presented. We introduce a statistical test on MFBM based on its ACVF which is presented as a quadratic form. Next, the power of the test is studied for the three cases corresponding to different Hurst exponent functions. We show the areas where the test is very strong in distinguishing between the processes and the cases when it fails in this respect. Finally, Section 4 summarizes and concludes our work.

## 2. Model and Methods

Let us start with a definition of the MFBM.

**Definition** **1.**  *(Multifractional Brownian motion). Process ${\left(B_{H(t)}\left(t\right)\right)}_{t\geq 0}$ is called a multifractional Brownian motion (MFBM) if it is a centered Gaussian process with covariance function*

(2) $$\begin{array}{cc} \mathrm{Cov}(B_{H(t)}\left(t\right),B_{H(s)}\left(s\right)) & =D\left(H\left(t\right),H\left(s\right)\right)\left(t^{H(t)+H(s)}+s^{H(t)+H(s)}-{\left|t-s\right|}^{H(t)+H(s)}\right),\\ where \end{array}$$

(3) $$\begin{array}{cc} D(x,y) & =\frac{\sigma^{2}\sqrt{\Gamma (2x+1)\Gamma (2y+1)\sin(\pi x)\sin(\pi y)}}{2\Gamma \left(x+y+1\right)\sin\left(\pi \frac{x+y}{2}\right)} \end{array}$$

*for some σ>0 and Hölder function $H:\;\left[0,\infty \right)\to \left[a,b\right]\subset \left(0,1\right)$ of some exponent β>0 [46].*

The second moment of MFBM scales as $E\left(B_{H(t)}^{2}\left(t\right)\right)=\sigma^{2}t^{2H(t)}$. Hence, we will call H(t) the Hurst exponent function. Furthermore, for H(t)≡H∈(0,1) MFBM becomes standard FBM. In general, MFBM has non-stationary increments. Its increment process $Y\left(t\right)\overset{def}{=}B_{H(t+1)}\left(t+1\right)-B_{H(t)}\left(t\right)$ possesses the long-range dependence property, in the sense that

$$\begin{array}{c} \forall \delta >0,\forall s\geq 0\;\sum_{k=0}^{\infty }\left|\mathrm{Corr}\left(Y(s),Y(s+k\delta )\right)\right|=+\infty , \end{array}$$

where Corr is the correlation function, i.e., $\mathrm{Corr}\left(Y\left(t\right),Y\left(s\right)\right)=\frac{\mathrm{Cov}(Y(t),Y(s))}{\sqrt{E^{2}Y\left(t\right)E^{2}Y\left(s\right)}}$ [46].

Furthermore, the function H(t) can be pointwise interpreted as a local self-similarity parameter, i.e.,

$$\begin{array}{c} \lim_{\varepsilon \to 0^{+}}{\left(\frac{B_{H(u+\varepsilon t)}\left(u+\varepsilon t\right)-B_{H(u)}\left(u\right)}{\varepsilon^{H(u)}}\right)}_{t\in R_{+}}=s\left(u\right){\left(B_{H}\left(t\right)\right)}_{t\in R_{+}}, \end{array}$$

where $B_{H}$ is a fractional Brownian motion with index H≡H(u), s(u) is a scaling function [47] and the convergence is on the space of continuous functions endowed with the topology of the uniform convergence on compact sets.

### 2.1. Mean-Squared Displacement

Let us now recall different estimators of the MSD for a sample of *n* trajectories $X_{1},X_{2},\ldots X_{n}$, each with *N* observations, that is, $X_{i}$ consists of $X_{i}\left(t_{1}\right),X_{i}\left(t_{2}\right),\ldots ,X_{i}\left(t_{N}\right)$ equally spaced in time, i=1,2,…,n. Ensemble average MSD (EAMSD) is defined as follows:

(4) $$\begin{array}{c} \mathrm{EAMSD}\left(\tau =m\Delta t\right)=\frac{1}{n}\sum_{k=1}^{n}{\left(X_{k}\left(t_{1}+\tau \right)-X_{k}\left(t_{1}\right)\right)}^{2}, \end{array}$$

where m=1,…,N and $\Delta t=t_{2}-t_{1}$. Time average MSD (TAMSD) is defined for each trajectory as

(5) $$\begin{array}{c} \mathrm{TAMSD}\left(\tau =m\Delta t,k\right)=\frac{1}{N-m}\sum_{j=1}^{N-m}{\left(X_{k}\left(t_{j}+m\Delta t\right)-X_{k}\left(t_{j}\right)\right)}^{2}. \end{array}$$

Finally, we consider ensemble and time average MSD (EATAMSD) which is an average of TAMSDs:

(6) $$\begin{array}{c} \mathrm{EATAMSD}\left(\tau \right)=\frac{1}{n}\sum_{k=1}^{n}\mathrm{TAMSD}\left(\tau ,k\right). \end{array}$$

Physicists often observe systems where EA and EATAMSD are different. Such behavior is called weak ergodicity breaking. In mathematics, the notion of ergodicity is restricted to stationary processes. Since the increments of MFBM lack stationarity, when analyzing the results, one has to be exceedingly meticulous.

### 2.2. Three Cases of the Hurst Exponent Function

Following [48], we consider three basic families of the function H(t), namely

$$\begin{array}{cc} \mathrm{linear}\; & H(t)=at+b,\;t\in [0,T],\\ \mathrm{logistic}\; & H\left(t\right)=\frac{c-b}{1+\exp\left\{-d\frac{t-t_{0}}{T}\right\}}+b,\;t\in \left[0,T\right],\\ \mathrm{periodic}\; & H\left(t\right)=a\sin\left(4\pi \frac{t}{T}\right)+b,\;t\in \left[0,T\right] \end{array}$$

for some time horizon T>0. Furthermore, in the sequel we consider only case with parameter $\sigma^{2}=1$ in (2). Such functions are continuous and as a consequence satisfy the Hölder condition, so in order to MFBM be properly defined we only require H(t)∈(0,1), for all t∈[0,T] [42]. Such choice of considered functions can be interpreted as follows. In the linear case, MFBM can switch steadily from short- to long-range dependence or vice versa, whereas in the logistic case such change is quite rapid and it happens between two levels. The latter case closely resembles instantaneous change in dependence or jump-type regime switching (such cases would lead to non-Hölder function). An alternative function to the logistic which is also considered is the literature is the arctan function [49]. Finally, the periodic case represents a situation where such changes are gradual and repetitive.

In the paper, we focus on the following special cases with specified parameters:

$$\begin{array}{cc} \mathrm{linear}\mathrm{function}:\; & H^{\left(1\right)}\left(t\right)=\frac{0.3}{1000}t+0.3,\;t\in \left[0,1000\right],\\ \mathrm{logistic}\mathrm{function}:\; & H^{\left(2\right)}\left(t\right)=\frac{0.3}{1+\exp\left\{-100\frac{t-500}{1000}\right\}}+0.3,\;t\in \left[0,1000\right],\\ \mathrm{periodic}\mathrm{case}:\; & H^{\left(3\right)}\left(t\right)=0.15\sin\left(4\pi \frac{t}{1000}\right)+0.45.\;t\in \left[0,1000\right]. \end{array}$$

**Figure 1:**
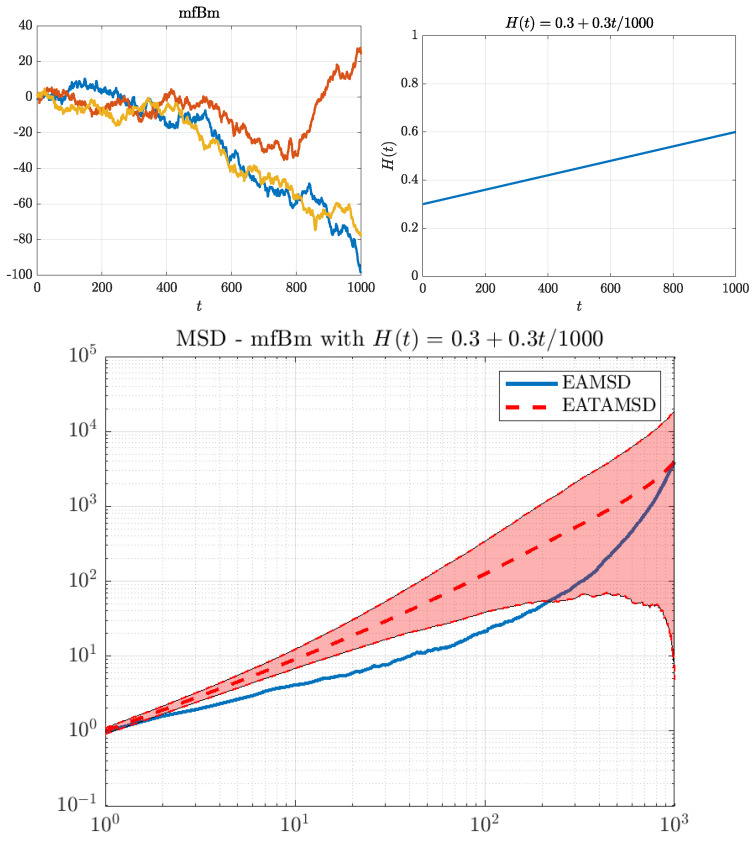
MFBM with the linear Hurst exponent function. Top left panel: three simulated trajectories. Top right panel: illustration of the function H(t) used in simulations. Bottom panel: comparison of EAMSD (solid blue line) with EATAMSD (dashed red line) and its 95% confidence interval (red shaded area). EAMSD and ETAMSD with confidence interval were calculated on the basis of 1000 simulated trajectories of MFBM.

**Figure 2:**
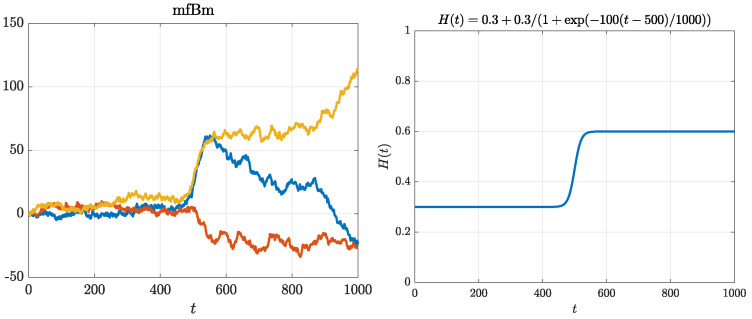

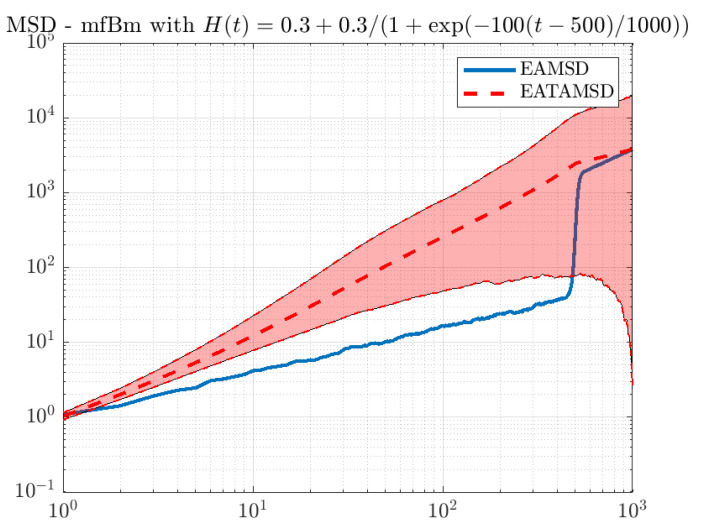
MFBM with the logistic Hurst exponent function. Top left panel: three simulated trajectories. Top right panel: illustration of the function H(t) used in simulations. Bottom panel: comparison of EAMSD (solid blue line) with EATAMSD (dashed red line) and its 95% confidence interval (red shaded area). EAMSD and ETAMSD with confidence interval were calculated on the basis of 1000 simulated trajectories of MFBM.

**Figure 3:**
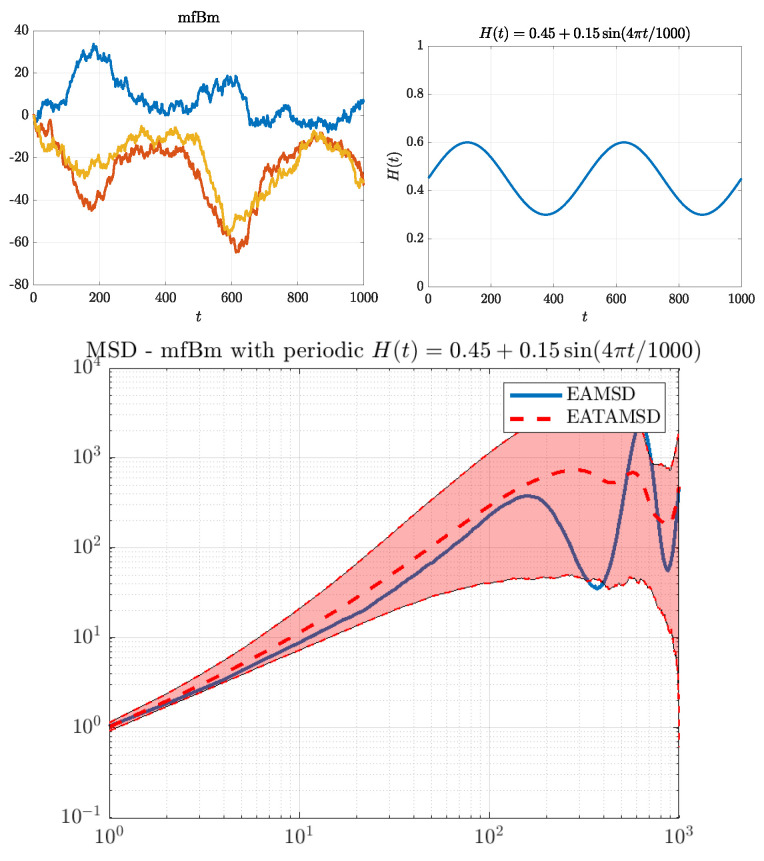
MFBM with the periodic Hurst exponent function function. Top left panel: three simulated trajectories. Top right panel: illustration of the function H(t) used in simulations. Bottom panel: comparison of EAMSD (solid blue line) with EATAMSD (dashed red line) and its 95% confidence interval (red shaded area). EAMSD and ETAMSD with confidence interval were calculated on the basis of 1000 simulated trajectories of MFBM.

We choose those specific parameters so that all of the cases have a similar “average” behavior of the function H(t), i.e., its mean is close to 0.45, and the function itself has values in the interval [0.3,0.6]. We illustrate those cases in Figure 1, Figure 2 and Figure 3. On the top left panel of each of these figures, we can see three simulated trajectories. The function H(t) is presented on the top right panel, whereas on the bottom panel we can see a behavior of the corresponding MSDs. It is important to note that EAMSD (blue line) is directly related to the variance of the model at time τ, i.e., $\mathrm{EAMSD}\left(\tau \right)=\hat{\mathrm{Var}}\left(X\left(\tau \right)\right)$, thus, from (2), it should behave like $\tau^{2H(\tau )}$.

For the linear case, see Figure 1, since $H^{\left(1\right)}\left(t\right)$ increases steadily from 0.3 to 0.6 we can see trajectories exhibit more variability for bigger times. This is also related to the EAMSD dynamics. In addition, such a model exhibits weak ergodicity breaking behavior (i.e., lack of equality between EATAMSD and EAMSD), which can be inferred from the bottom panel.

Next, for the logistic case, see Figure 2, $H^{\left(2\right)}\left(t\right)$ increases quite rapidly from 0.3 to 0.6 near t=500. As a consequence, we can see a switch in the behavior of simulated trajectories: for times t<500 they exhibit far less variability than for t>500. Intuitively, for times t<500 trajectories locally exhibit short-range dependence, whereas for t>500 they locally exhibit long-range dependence. Again, we can see weak ergodicity breaking on the bottom panel.

Finally, for the periodic case, see Figure 3, $H^{\left(3\right)}\left(t\right)$ varies between 0.3 and 0.6. We can clearly see two different regimes of behavior: for times when $H^{\left(3\right)}\left(t\right)$ is bigger, trajectories are smoother and generally have larger values, in contrast to times when $H^{\left(3\right)}\left(t\right)$ is smaller. Despite lack of stationarity, here, EAMSD almost always lies in the confidence region of EATAMSD, which could suggest there is no weak ergodicity breaking.

## 3. Results

In applications, it is crucial to be able to check whether a stochastic model describes empirical data well. Despite dedicated identification methods for the MFBM [50,51,52,53], to the best of the authors’ knowledge, there is no rigorous statistical test designed for such process. Here, we propose an approach using a simple test statistic which also contains useful information about the process itself.

### 3.1. Test

For the testing purposes, we follow an approach based on the ACVF which was introduced by Balcerek and Burnecki [38]. ACVF is a very popular statistic and it is also one of the simplest quadratic forms. For a random sample $X_{N}=\left\{X\left(1\right),X\left(2\right),\ldots ,X\left(N\right)\right\}$ and τ∈{1,2,⋯,N−1}, it is defined as follows:

(7) $$\begin{array}{c} {\mathrm{ACVF}}_{N}\left(\tau \right)=\frac{1}{N-\tau }\sum_{i=1}^{N-\tau }X\left(i+\tau \right)X\left(i\right). \end{array}$$

Here, we only consider a version of ACVF without subtracting the sample mean as it does not influence performance of tests based on this statistic for a centered process [38] and it makes the formulas much simpler.

Let us now introduce a matrix $A\left(\tau \right)=\{a{\left(\tau ;i,j)\right\}}_{i,j=1}^{N}$, where

(8) $$\begin{array}{c} a\left(\tau ;i,j\right)=\left\{\begin{array}{cc} \frac{1}{N}I\left(i=j\right) & \mathrm{if}\tau =0\\ \frac{1}{2}\frac{1}{N-\tau }I\left(|i-j|=\tau \right) & \mathrm{if}\tau =1,2,\ldots ,N-1\\ 0 & \mathrm{otherwise}, \end{array}\right. \end{array}$$

and I is the indicator. To summarize, the matrix A(τ) is either diagonal (for τ=0) with elements $\frac{1}{N}$ on diagonal or Toeplitz, with only two nonzero subdiagonals (starting at (1+τ)th row and (1+τ)th column) with elements $\frac{1}{2}\frac{1}{N-\tau }$. The statistic ${\mathrm{ACVF}}_{N}$ can be now expressed as a quadratic form (as shown in [38]) as a generalized $\chi^{2}$ distribution, that is

(9) $$\begin{array}{c} {\mathrm{ACVF}}_{N}\left(\tau \right)=\sum_{i=1}^{N}\lambda_{i}\left(\tau \right)Z_{i}^{2}, \end{array}$$

where $Z_{i}$s are i.i.d standard normal variables (so $Z_{i}^{2}$ has a $\chi_{1}^{2}$ distribution) and $\lambda_{k}\left(\tau \right)$ are eigenvalues of the matrix $\Sigma_{N}\left(\tau \right)=\Sigma^{1/2}A\left(\tau \right)\Sigma^{1/2}$ with Σ being the (theoretical) autocovariance matrix of our trajectory $X_{N}$. It is important to note that this result is true regardless of whether the considered model is stationary or not.

Let us now formulate a test for checking whether a random sample $X_{N}$ comes from the MFBM with function H:[0,T]→(0,1), where *T* is the time horizon:

$$\begin{array}{cc} \; & H_{0}:\mathrm{sample}\mathrm{comes}\mathrm{from}\mathrm{the}\mathrm{model}\mathrm{with}\mathrm{function}H\left(t\right)\\ \; & \mathrm{versus}\\ \; & H_{1}:\mathrm{sample}\mathrm{comes}\mathrm{from}\mathrm{the}\mathrm{model}\mathrm{with}\mathrm{function}\mathrm{different}\mathrm{than}H\left(t\right). \end{array}$$

We will use ${\mathrm{ACVF}}_{N}$ as a test statistic with its distribution given by Equation (9) to calculate critical regions of such test for a given significance level. Naturally, eigenvalues $\lambda_{i}\left(\tau \right)$ depend on the matrix A(τ) as well as on the matrix Σ. Elements of Σ are given by ACVF (2) and are calculated using the function H(t) from the null hypothesis.

### 3.2. Three Power Case Studies

The power of the test is the probability to reject the null hypothesis when the alternative is true. The power is an important characteristic of any statistical test. We consider the following null hypotheses, which correspond to the examples presented in Figure 1, Figure 2 and Figure 3.

$$\begin{array}{cc} \mathrm{linear}\;\mathrm{function}\;\mathrm{null}\;\mathrm{hypothesis}\;H_{0}:\; & H\left(t\right)=H^{\left(1\right)}\left(t\right)\;t\in \left[0,1000\right],\\ \mathrm{logistic}\;\mathrm{function}\;\mathrm{null}\;\mathrm{hypothesis}\;H_{0}:\; & H\left(t\right)=H^{\left(2\right)}\left(t\right),\;t\in \left[0,1000\right],\\ \mathrm{periodic}\;\mathrm{case}\;\mathrm{null}\;\mathrm{hypothesis}\;H_{0}:\; & H\left(t\right)=H^{\left(3\right)}\left(t\right),\;t\in \left[0,1000\right]. \end{array}$$

In our studies, for all considered cases, we calculate the power of the test by using Monte Carlo simulations. We assume that the significance level is equal to 5%. In our Monte Carlo simulations we consider the time horizon T=1000 and equally spaced time points t=1,2,…,1000. For each set of parameters from the alternative hypothesis, we simulate n=1000 trajectories, calculate test statistic (7), and check if the null hypothesis is rejected at 5% significance level. In the test statistic, we consider only τ=1 since other choices of τ lead to worse results. Finally, we estimate the power of this test for each considered case by calculating the fraction of rejected null hypotheses.

We present the results in the form of power functions with arguments being the parameters of the function H(t) from the alternative hypothesis. For all of the cases, we considered the alternative coming from the same family of functions as the function H(t) in the null hypothesis, i.e., linear alternative for linear null, etc.

**Figure 4:**
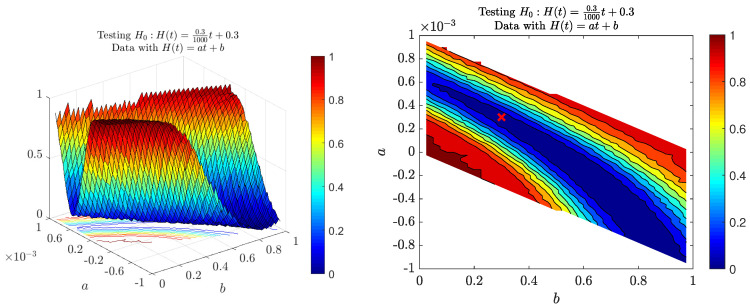
Power of the introduced test for the linear Hurst exponent function H(t)=at+b with respect to parameters *a* and *b*. The null hypothesis is $a=\frac{0.3}{1000}$ and b=0.3. The right panel depicts the results in the form of a heat map with the red ‘x’ sign representing parameters in the null hypothesis. White regions represent areas for which MFBM is not well defined. The powers were calculated by means of Monte Carlo simulations on the basis of simulated data from the MFBM with different *a*s and *b*s.

First, let us consider testing MFBM with the linear Hurst exponent function. We can see the power function related to that case in Figure 4. The left panel presents the power function with respect to parameters *a* and *b* from the alternative hypothesis $H_{1}:H\left(t\right)=at+b$, the right panel the corresponding heat map. We can see “layers” (regions on the heat map with the same color) for which our test has a very similar power. For example, the deep blue region on the right panel corresponds to the processes indistinguishable from the null hypothesis process. We believe that the shape of the region is related to the construction of our test, namely our test statistic ${\mathrm{ACVF}}_{N}$ takes into account all addends X(t)X(t+τ) with the same weight, thus it is not that relevant whether *t* is big or not. In the case of MFBM, for which neither the process nor its increments are stationary, it might be an important factor. As a consequence, we can see that the test has a difficulty in distinguishing between MFBMs with increasing and decreasing Hurst exponent functions if their means are similar. However, this conjecture is not very precise, namely the mean case H≡0.45, which matches the alternative hypothesis with a=0,b=0.45, yields a much higher power of the test than the significance level. We also note that for parameters from the null hypothesis: a=0.3,b=0.3 power of such test is approximately equal to 5%, which is the assumed significance level. On the heat map, white regions represent areas for which the process MFBM is not well-defined, i.e., H(t)∉(0,1) for some t∈[0,1000].

**Figure 5:**
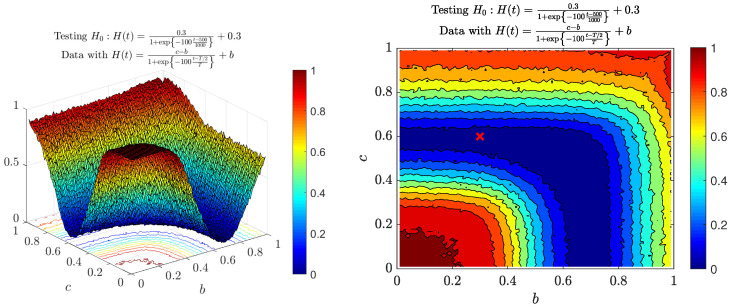
Power of the introduced test for the logistic Hurst exponent function $H\left(t\right)=\frac{c-b}{1+\exp\left\{-100\frac{t-500}{1000}\right\}}+b$ with respect to parameters *c* and *b*. The null hypothesis is c=0.6 and b=0.3. The right panel depicts the results in the form of heat map with the red ‘x’ sign representing parameters in the null hypothesis. White regions represent areas for which MFBM is not well defined. The powers were calculated by means of Monte Carlo simulations on the basic of simulated data from the MFBM with different *c*s and *b*s.

Let us now consider the second case, that is MFBM with the logistic function H(t). We can observe the power function in Figure 5. Left panel presents the power function with respect to parameters *b* and *c* from the alternative hypothesis $H_{1}:H\left(t\right)=\frac{c-b}{1+\exp\left\{-d\frac{t-t_{0}}{T}\right\}}+b$, the right panel the corresponding heat map. The parameter *b* is related to the local self-similarity parameter for t<500, and *c* for t>500. Similarly to the case with the linear function null hypothesis, here we can observe “layers” in which parameters *b* and *c* are almost symmetric (e.g., the null hypothesis case where b=0.3 and c=0.6 is closely related to the case b=0.6 and c=0.3). Moreover, we can see that the power function seems to be quite high in the cases when a tested sample has the *b* parameter close to the value 0.3 from the null hypothesis, but *c* is far from 0.6, or vice versa.

**Figure 6:**
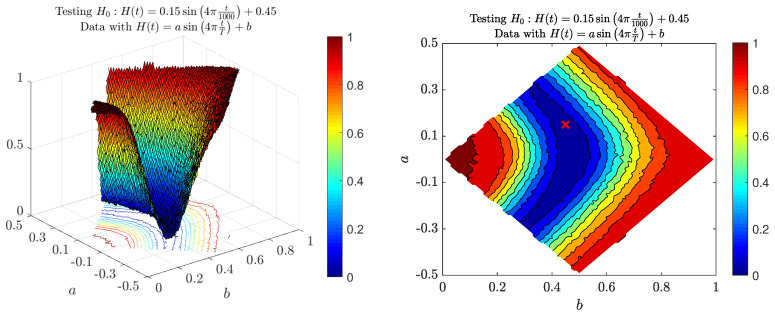
Power of the introduced test for the periodic Hurst exponent function $H\left(t\right)=a\sin\left(4\pi \frac{t}{1000}\right)+b$ with respect to parameters *a* and *b*. The null hypothesis is a=0.15 and b=0.45. The right panel depicts the results in the form of heat map with the red ‘x’ sign representing parameters in the null hypothesis. White regions represent areas for which MFBM is not well defined. The powers were calculated by means of Monte Carlo simulations on the basic of simulated data from the MFBM with different *a*s and *b*s.

Lastly, let us consider the case of MFBM with the periodic function H(t). We can observe the power function in Figure 6. The left panel presents the power function with respect to parameters *a* and *b* from the alternative hypothesis $H_{1}:H\left(t\right)=a\sin\left(4\pi \frac{t}{T}\right)+b$, the right panel the corresponding heat map. Parameter *b* is related to the “mean” behavior of the function H(t), whereas the parameter *a* corresponds to its amplitude. Again, similarly to the two previous null hypotheses, we can observe “layers” of similar power values. Those layers are symmetric with respect to a=0. This means that for alternatives with opposite parameters *a* the test seems to return the same power. Let us note that this is not intuitive, namely such opposite *a*s are related to completely different local behaviors of the self-similarity parameter. On the heat map, white regions represent areas for which process MFBM is not well defined, i.e., H(t)∉(0,1) for some t∈[0,1000].

Finally, we would like to emphasize that the introduced test requires MFBM parameters to be fixed (we test if the data follow MFBM with fixed parameters). In practice, when analyzing empirical data the parameters are often estimated. In the literature, methods for estimation of the Hurst exponent function H(t) in the MFBM framework have been already introduced [50,51,52] and later combined to improve both the goodness of fit and the computational speed of the algorithm [53].

## 4. Discussion and Conclusions

For power-law anomalous diffusion of the form (1) with constant anomalous diffusion exponent α a number of models exist, including continuous-time random walks, fractional Langevin equation motion, FBM, or scaled Brownian motion [8]. These models all have different physical properties such as the PDF or their ergodic and aging properties [8].

In this paper, we concentrated on MFBM which is a generalization of FBM for Hölder continuous functions H(t) that allows the Hurst exponent to vary in time. The time-varying Hurst exponent has an impact on both the statistical properties of the process and trajectory characteristics. MFBM helps to model phenomena whose regularity of paths and anomalous diffusion exponent change in time. The process has no longer stationary increments and it is not self-similar but the variance scales in a natural way as $t^{2H(t)}$.

Following the idea of testing FBM based on the ACVF statistic [38], in this paper, we introduced a rigorous statistical test on MFBM with the ACVF statistic presented as a quadratic form. We derived the distribution of the statistic which is the generalized $\chi^{2}$. In order to study the efficiency of the test, we took into consideration three possible classes of the Hurst exponent function, namely linear, logistic, and periodic. For those cases, we conducted power studies with the help of Monte Carlo simulations. As alternatives, we considered MFBMs within the same class of the Hurst exponent function but with different parameters.

We found ranges of the parameters where the test is more sensitive to differences and ranges where it fails to distinguish between the models. It appears that for the linear Hurst exponent function the test is most sensitive to changes in the mean of the function. If the means are similar then the test often fails, even if the functions have completely different patterns, namely, one is increasing and the other decreasing. The latter observation may sound like a serious objection for using the test, but, in practice, an experimentalist knows whether the anomalous diffusion exponent increases or decreases in time. In the logistic case, the situation is different, namely the mean does not matter much as for the linear case. Now, the test is most sensitive to deviations from the true values of the two levels (*c* and *b*) with the exception that replacing *c* with *b* does not change the power of the test (so, again, it does not matter if the function increases or decreases). For the periodic case, we have again a different situation. The test is sensitive to the changes of the amplitude of the sine function and the value of the free term but it does not detect a sign of the parameter related to the magnitude.

**Figure A1:**
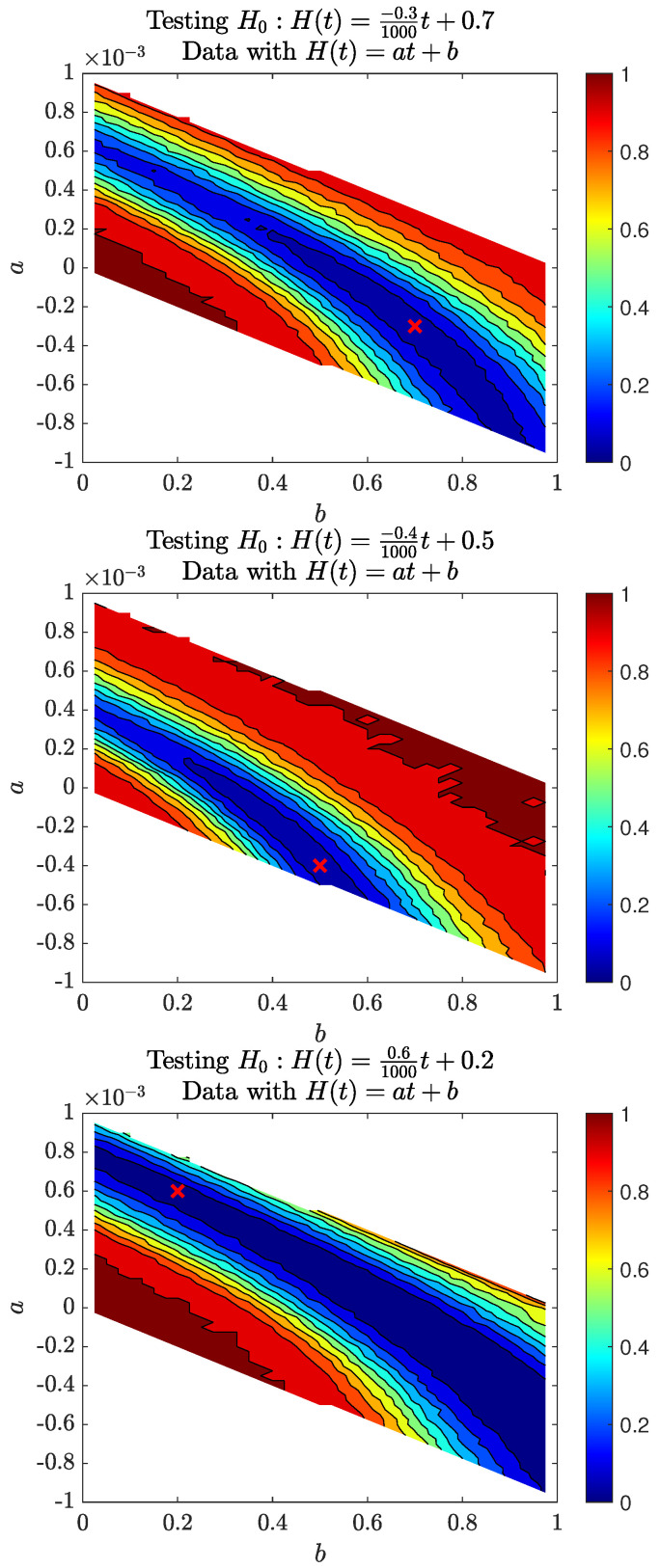
Power of the introduced test for the linear Hurst exponent function H(t)=at+b with respect to parameters *a* and *b*. The null hypotheses are: $a=\frac{-0.3}{1000}$ and b=0.7 (top panel), $a=\frac{-0.4}{1000}$ and b=0.5 (middle panel), $a=\frac{0.6}{1000}$ and b=0.2 (bottom panel). All of the panels depict the results in the form of a heat map with the red ‘x’ sign representing parameters in the null hypothesis. White regions represent areas for which MFBM is not well defined. The powers were calculated by means of Monte Carlo simulations on the basis of simulated data from the MFBM with different *a*s and *b*s.

**Figure A2:**
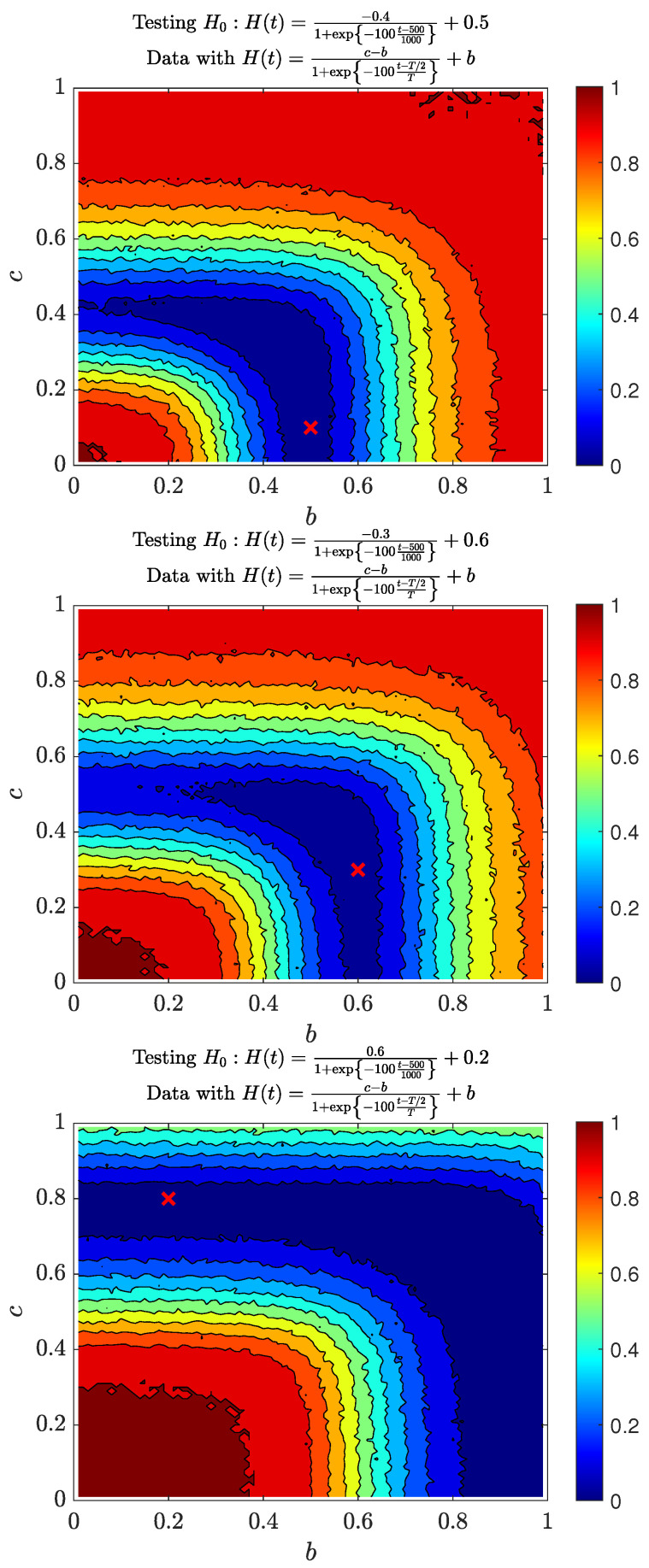
Power of the introduced test for the logistic Hurst exponent function $H\left(t\right)=\frac{c-b}{1+\exp\left\{-100\frac{t-500}{1000}\right\}}+b$ with respect to parameters *c* and *b*. The null hypotheses are: c=0.1 and b=0.5 (top panel), c=0.3 and b=0.6 (middle panel), c=0.8 and b=0.2 (bottom panel). All of the panels depict the results in the form of heat map with the red ‘x’ sign representing parameters in the null hypothesis. White regions represent areas for which MFBM is not well defined. The powers were calculated by means of Monte Carlo simulations on the basic of simulated data from the MFBM with different *c*s and *b*s.

**Figure A3:**
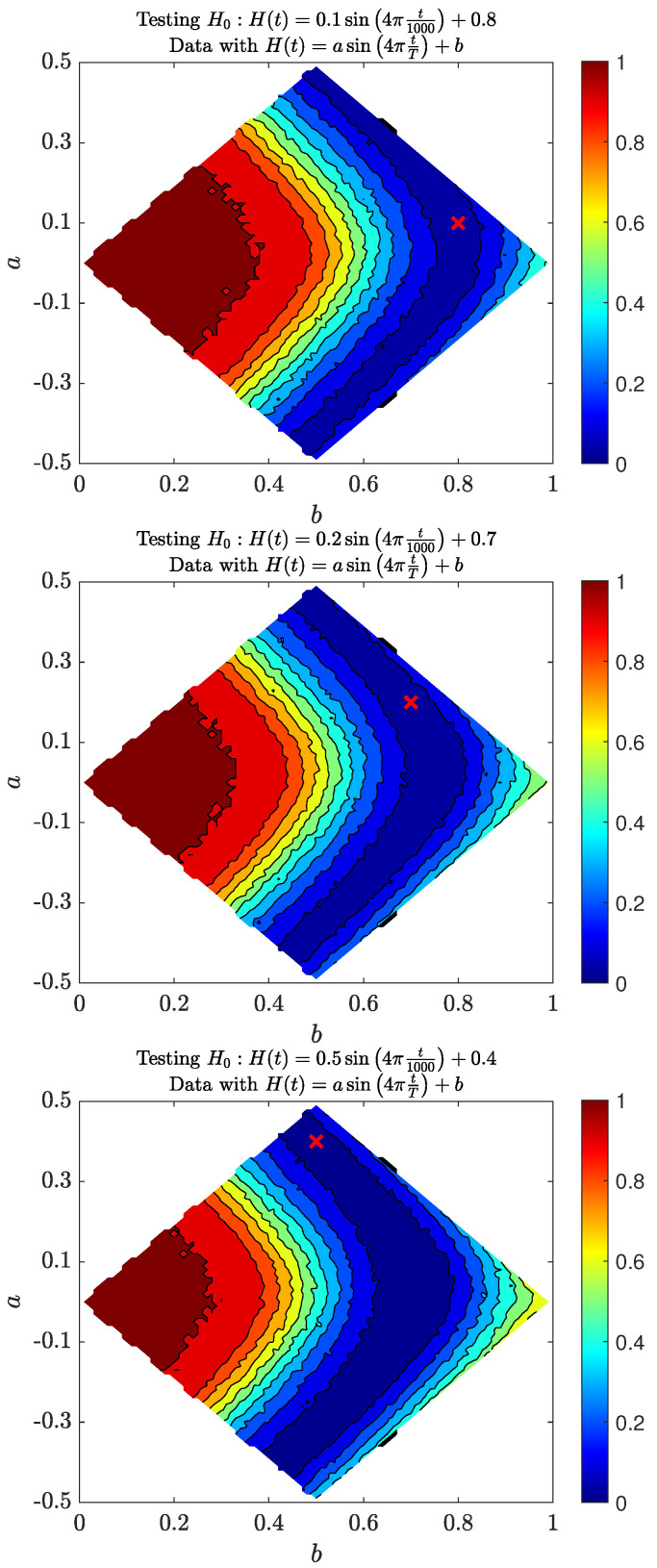
Power of the introduced test for the periodic Hurst exponent function $H\left(t\right)=a\sin\left(4\pi \frac{t}{1000}\right)+b$ with respect to parameters *a* and *b*. The null hypotheses are: a=0.1 and b=0.8 (top panel), a=0.2 and b=0.7 (middle panel), a=0.5 and b=0.4 (bottom panel). All of the panels depict the results in the form of heat map with the red ‘x’ sign representing parameters in the null hypothesis. White regions represent areas for which MFBM is not well defined. The powers were calculated by means of Monte Carlo simulations on the basic of simulated data from the MFBM with different *a*s and *b*s.

Finally, we note that we checked the behavior of the test for other sets of parameters of the null hypotheses and different sample lengths, and the conclusions were similar. We only found that the range of possible *H* values from the null hypothesis has an influence on the width of the acceptance regions (the wider the range the wider the acceptance region, which is reasonable). We present some of the additional tests’ power simulation studies in Appendix A. Figure A1 presents different cases of the null hypothesis for the linear case, Figure A2 for the logistic case, and Figure A3 for the periodic case. Tests for the linear case were performed for length N=1000, whereas logistic and periodic cases were studied for length N=200.

In sum, we introduced a rigorous statistical test for MFBM based on ACVF statistic presented as a quadratic form. We highlighted the weak and strong points of the test. Improving the efficiency of the test will be a subject of our future studies. We believe that the obtained results can help to understand the mechanisms underlying various anomalous diffusion phenomena.

## Abbreviations

The following abbreviations are used in this manuscript:

SPT	Single-particle tracking
FBM	Fractional Brownian motion
MFBM	Multifractional Brownian motion
MSD	Mean-squared displacement
TAMSD	Time average mean-squared displacement
EAMSD	Ensemble average mean-squared displacement
EATAMSD	Ensemble and time average mean-squared displacement
ACVF	Autocovariance function

## Appendix A

In Figure A1–Figure A3, we present power functions of tests related to different null hypotheses. In Figure A1, we consider $H_{0}:H\left(t\right)=\frac{-0.3}{1000}t+0.7$ (top panel), so a case in which function *H* begins in the superdiffusive regime and then decreases linearly to value 0.4; $H_{0}:H\left(t\right)=\frac{-0.4}{1000}t+0.5$ (middle panel), so a case in which function *H* begins in the diffusion regime and then decreases linearly to value 0.1; and $H_{0}:H\left(t\right)=\frac{0.6}{1000}t+0.2$ (bottom panel), so a case in which function *H* begins in the strong subdiffusive regime and then increases linearly to 0.8.

In Figure A2, we consider $H_{0}:H\left(t\right)=\frac{-0.4}{1+\exp\left\{-100\frac{t-500}{1000}\right\}}+0.5$ (top panel), so a case in which function *H* begins in the diffusive regime and ends in the strong subdiffusive regime; $H_{0}:H\left(t\right)=\frac{-0.3}{1+\exp\left\{-100\frac{t-500}{1000}\right\}}+0.6$ (middle panel), so a case in which function *H* begins in the superdiffusive regime and ends in the subdiffusive regime; and $H_{0}:H\left(t\right)=\frac{0.6}{1+\exp\left\{-100\frac{t-500}{1000}\right\}}+0.2$ (bottom panel), so a case in which function *H* begins in the strong subdiffusive regime and ends in the strong superdiffusive regime.

In Figure A3, we consider $H_{0}:H\left(t\right)=0.1\sin\left(4\pi \frac{t}{1000}\right)+0.8$ (top panel), so a case in which function *H* varies periodically between 0.7 and 0.9, i.e., in the strong superdiffusion regime; $H_{0}:H\left(t\right)=0.2\sin\left(4\pi \frac{t}{1000}\right)+0.7$ (middle panel), so a case in which function *H* varies periodically between 0.5 and 0.9, i.e., in the superdiffusion regime; and $H_{0}:H\left(t\right)=0.5\sin\left(4\pi \frac{t}{1000}\right)+0.4$ (bottom panel), so a case in which function *H* varies periodically between 0.1 and 0.9, i.e., in the whole spectrum of anomalous diffusion.

## References

[1] Braüchle C., Lamb D.C., Michaelis J. (2010). Single Particle Tracking and Single Molecule Energy Transfer.

[2] Xie X.S., Choi P.J., Li G.W., Lee N.K., Lia G. (2008). Single-molecule approach to molecular biology in living bacterial cells. Annu. Rev. Biophys..

[3] Metzler R., Jeon J.H., Cherstvy A. (2016). Non-Brownian diffusion in lipid membranes: Experiments and simulations. Biochim. Biophys. Acta (BBA)-Biomembr..

[4] Saxton M.J., Jacobson K. (1997). Single-particle tracking: Applications to membrane dynamics. Annu. Rev. Biophys. Biomol. Struct..

[5] Bouchaud J.P., Georges A. (1990). Anomalous diffusion in disordered media: Statistical mechanisms, models and physical applications. Phys. Rep..

[6] Höfling F., Franosch T. (2013). Anomalous transport in the crowded world of biological cells. Rep. Prog. Phys..

[7] Barkai E., Garini Y., Metzler R. (2012). Strange kinetics of single molecules in living cells. Phys. Today.

[8] Metzler R., Jeon J.H., Cherstvy A.G., Barkai E. (2014). Anomalous diffusion models and their properties: Non-stationarity, non-ergodicity, and ageing at the centenary of single particle tracking. Phys. Chem. Chem. Phys..

[9] Golding I., Cox E.C. (2006). Physical nature of bacterial cytoplasm. Phys. Rev. Lett..

[10] Bronstein I., Israel Y., Kepten E., Mai S., Shav-Tal Y., Barkai E., Garini Y. (2009). Transient anomalous diffusion of telomeres in the nucleus of mammalian cells. Phys. Rev. Lett..

[11] Jeon J.H., Tejedor V., Burov S., Barkai E., Selhuber-Unkel C., Berg-Sørensen K., Oddershede L., Metzler R. (2011). In vivo anomalous diffusion and weak ergodicity breaking of lipid granules. Phys. Rev. Lett..

[12] Weigel A.V., Simon B., Tamkun M.M., Krapf D. (2011). Ergodic and nonergodic processes coexist in the plasma membrane as observed by single-molecule tracking. Proc. Natl. Acad. Sci. USA.

[13] Tabei S.A., Burov S., Kim H.Y., Kuznetsov A., Huynh T., Jureller J., Philipson L.H., Dinner A.R., Scherer N.F. (2013). Intracellular transport of insulin granules is a subordinated random walk. Proc. Natl. Acad. Sci. USA.

[14] Szymanski J., Weiss M. (2009). Elucidating the origin of anomalous diffusion in crowded fluids. Phys. Rev. Lett..

[15] Jeon J.H., Leijnse N., Oddershede L.B., Metzler R. (2013). Anomalous diffusion and power-law relaxation of the time averaged mean squared displacement in worm-like micellar solutions. New J. Phys..

[16] Wong I., Gardel M., Reichman D., Weeks E.R., Valentine M., Bausch A., Weitz D.A. (2004). Anomalous diffusion probes microstructure dynamics of entangled F-actin networks. Phys. Rev. Lett..

[17] Hansing J., Ciemer C., Kim W.K., Zhang X., DeRouchey J.E., Netz R.R. (2016). Nanoparticle filtering in charged hydrogels: Effects of particle size, charge asymmetry and salt concentration. Eur. Phys. J. E.

[18] Xu Q., Feng L., Sha R., Seeman N., Chaikin P. (2011). Subdiffusion of a sticky particle on a surface. Phys. Rev. Lett..

[19] Godec A., Bauer M., Metzler R. (2014). Collective dynamics effect transient subdiffusion of inert tracers in flexible gel networks. New J. Phys..

[20] Weiss M., Hashimoto H., Nilsson T. (2003). Anomalous protein diffusion in living cells as seen by fluorescence correlation spectroscopy. Biophys. J..

[21] Kneller G.R., Baczynski K., Pasenkiewicz-Gierula M. (2011). Communication: Consistent picture of lateral subdiffusion in lipid bilayers: Molecular dynamics simulation and exact results. J. Chem. Phys..

[22] Jeon J.H., Monne H.M.S., Javanainen M., Metzler R. (2012). Anomalous diffusion of phospholipids and cholesterols in a lipid bilayer and its origins. Phys. Rev. Lett..

[23] Yamamoto E., Akimoto T., Yasui M., Yasuoka K. (2014). Origin of subdiffusion of water molecules on cell membrane surfaces. Sci. Rep..

[24] Manzo C., Torreno-Pina J.A., Massignan P., Lapeyre G.J., Lewenstein M., Parajo M.F.G. (2015). Weak ergodicity breaking of receptor motion in living cells stemming from random diffusivity. Phys. Rev. X.

[25] Jeon J.H., Javanainen M., Martinez-Seara H., Metzler R., Vattulainen I. (2016). Protein crowding in lipid bilayers gives rise to non-Gaussian anomalous lateral diffusion of phospholipids and proteins. Phys. Rev. X.

[26] Berkowitz B., Cortis A., Dentz M., Scher H. (2006). Modeling non-Fickian transport in geological formations as a continuous time random walk. Rev. Geophys..

[27] Caspi A., Granek R., Elbaum M. (2000). Enhanced diffusion in active intracellular transport. Phys. Rev. Lett..

[28] Reverey J.F., Jeon J.H., Bao H., Leippe M., Metzler R., Selhuber-Unkel C. (2015). Superdiffusion dominates intracellular particle motion in the supercrowded cytoplasm of pathogenic Acanthamoeba castellanii. Sci. Rep..

[29] Gal N., Weihs D. (2010). Experimental evidence of strong anomalous diffusion in living cells. Phys. Rev. E.

[30] Monserud J.H., Schwartz D.K. (2016). Interfacial molecular searching using forager dynamics. Phys. Rev. Lett..

[31] Campagnola G., Nepal K., Schroder B.W., Peersen O.B., Krapf D. (2015). Superdiffusive motion of membrane-targeting C2 domains. Sci. Rep..

[32] Javanainen M., Hammaren H., Monticelli L., Jeon J.H., Miettinen M.S., Martinez-Seara H., Metzler R., Vattulainen I. (2013). Anomalous and normal diffusion of proteins and lipids in crowded lipid membranes. Faraday Discuss..

[33] Kolmogorov A.N. (1940). Wienersche Spiralen und einige andere interessante Kurven in Hilbertscen Raum. C.R. (Dokl.) Acad. Sci. URSS (NS).

[34] Mandelbrot B.B., Van Ness J.W. (1968). Fractional Brownian motions, fractional noises and applications. SIAM Rev..

[35] Graves T., Gramacy R., Watkins N., Franzke C. (2017). A brief history of long memory: Hurst, Mandelbrot and the road to ARFIMA, 1951–1980. Entropy.

[36] Goychuk I. (2012). Viscoelastic subdiffusion: Generalized Langevin equation approach. Adv. Chem. Phys..

[37] Klafter J., Lim S., Metzler R. (2012). Fractional Dynamics: Recent Advances.

[38] Balcerek M., Burnecki K. (2020). Testing of fractional Brownian motion in a noisy environment. Chaos Solitons Fractals.

[39] Sikora G., Burnecki K., Wyłomańska A. (2017). Mean-squared displacement statistical test for fractional Brownian motion. Phys. Rev. E.

[40] Sikora G. (2018). Statistical test for fractional Brownian motion based on detrending moving average algorithm. Chaos Solitons Fractals.

[41] Ralchenko K., Shevchenko G. (2010). Path properties of multifractal Brownian motion. Theory Probab. Math. Stat..

[42] Peltier R.F., Véhel J.L. (1995). Multifractional Brownian Motion: Definition and Preliminary Results.

[43] Lee K. (2013). Characterization of turbulence stability through the identification of multifractional Brownian motions. Nonlinear Process. Geophys..

[44] Perrin E., Harba R., Iribarren I., Jennane R. (2005). Piecewise fractional Brownian motion. IEEE Trans. Signal Process..

[45] Ryvkina J. (2015). Fractional Brownian Motion with variable Hurst parameter: Definition and properties. J. Theor. Probab..

[46] Ayache A., Cohen S., Véhel J.L. The covariance structure of multifractional Brownian motion, with application to long range dependence. Proceedings of the 2000 IEEE International Conference on Acoustics, Speech, and Signal Processing.

[47] Benassi A., Cohen S., Istas J. (1998). Identifying the multifractional function of a Gaussian process. Stat. Probab. Lett..

[48] Chan G., Wood A.T. (1998). Simulation of multifractional Brownian motion. COMPSTAT.

[49] Stoev S.A., Taqqu M.S. (2006). How rich is the class of multifractional Brownian motions?. Stoch. Process. Appl..

[50] Bianchi S. (2005). Pathwise identification of the memory function of multifractional Brownian motion with application to finance. Int. J. Theor. Appl. Financ..

[51] Bardet J.M., Bertrand P. (2007). Identification of the multiscale fractional Brownian motion with biomechanical applications. J. Time Ser. Anal..

[52] Bianchi S., Pantanella A., Pianese A. (2013). Modeling stock prices by multifractional Brownian motion: An improved estimation of the pointwise regularity. Quant. Financ..

[53] Pianese A., Bianchi S., Palazzo A.M. (2018). Fast and unbiased estimator of the time-dependent Hurst exponent. Chaos Interdiscip. J. Nonlinear Sci..

